# The Impact of CXCR4 Blockade on the Survival of Rat Brain Cortical Neurons

**DOI:** 10.3390/ijms17122005

**Published:** 2016-11-30

**Authors:** José Joaquín Merino, Alba Garcimartín, María Elvira López-Oliva, Juana Benedí, María Pilar González

**Affiliations:** 1 Departamento de Bioquímica y Biología Molecular II, Facultad de Farmacia, Universidad Complutense de Madrid (U.C.M.), Ciudad Universitaria, 28040 Madrid, Spain; pilarg@ucm.es; 2 Instituto de Investigación Neuroquímica (I.U.I.N.), Universidad Complutense de Madrid (U.C.M.), Ciudad Universitaria, 28040 Madrid, Spain; 3 Departamento de Farmacología, Facultad de Farmacia, Universidad Complutense de Madrid (U.C.M.), Ciudad Universitaria, 28040 Madrid, Spain; a.garcimartin@ucm.es (A.G.); jbenedi@ucm.es (J.B.); 4 Sección Departamental de Fisiología, Facultad de Farmacia, Universidad Complutense de Madrid (U.C.M.), Ciudad Universitaria, 28040 Madrid, Spain; elopez@ucm.es

**Keywords:** AMD3100, CXCR4 chemokine receptor, cortical neurons in vitro, caspase-3/9, apoptosis, cytochrome c/Bcl-2/Bax, mitochondria, cytosol, neuroprotection

## Abstract

Background: Chemokine receptor type 4 (CXCR4) plays a role in neuronal survival/cell repair and also contributes to the progression of cancer and neurodegenerative diseases. Chemokine ligand 12 (CXCL12) binds to CXCR4. In this study, we have investigated whether CXCR4 blockade by AMD3100 (a CXCR4 antagonist, member of bicyclam family) may affect neuronal survival in the absence of insult. Thus, we have measured the mitochondrial membrane potential (MMP), Bax and Bcl-2 protein translocation, and cytochrome c release in AMD3100-treated brain cortical neurons at 7 DIV (days in vitro). Methods: For this aim, AMD3100 (200 nM) was added to cortical neurons for 24 h, and several biomarkers like cell viability, reactive oxygen species (ROS) generation, lactate dehydrogenase (LDH) release, caspase-3/9 activity, proteins Bax and Bcl-2 translocation, and cytochrome *c* release were analyzed by immunoblot. Results: CXCR4 blockade by AMD3100 (200 nM, 24 h) induces mitochondrial hyperpolarization and increases caspase-3/9 hyperpolarization without affecting LDH release as compared to untreated controls. AMD3100 also increases cytochrome c release and promotes Bax translocation to the mitochondria, whereas it raises cytosolic Bcl-2 levels in brain cortical neurons. Conclusion: CXCR4 blockade induces cellular death via intrinsic apoptosis in rat brain cortical neurons in absence of insult.

## 1. Introduction

Chemokine receptor type 4 (CXCR4) is a seven-transmembrane G-α-chemokine-coupled receptor (GPCR) [1] specific for stromal-derived-factor-1 (SDF-1α), also called CXCL12 (chemokine ligand 12, its ligand). The CXCR4/SDF-1α axis contributes to inflammation, angiogenesis, tumorigenesis, neurodevelopment, and central nervous system (CNS) repair, among other processes [2,3,4,5]. CXCR4 function can be blocked by its antagonist, AMD3100 (bicyclam or plerixafor), a small molecule that contains two 1,4,8,11-tetraazacy-clotetradecane moieties [1].

Neurons express a variety of chemokine receptors, including CXCR4, that regulate neuronal signaling and survival [6]. Chemokines were recognized originally for their ability to dictate the migration and activation of leukocytes. CXCR4 receptors are present in newly generated neurons during embryogenesis and in mature neurons; they play a role in neurogenesis, neuronal guidance, and connectivity [6,7,8,9]. The levels of the receptors usually decrease as neurons mature. CXCR4-mutant mice have aberrant neuronal distribution in brain [10]. These findings highlight the diverse role of chemokines and their receptors (including CXCR4) in normal brain physiology (in the absent of insults).

The available data that report the effect of neuronal CXCR4 activation by SDF-1α on viability and function of neurons subjected to various insults are contradictory. For example, it was shown that in hippocampal or cerebellar granule neurons and in cell lines, the functional loss of cell cycle retinoblastoma protein (Rb) is associated with apoptosis. Exposure to SDF-1α rescues these cells from apoptosis and induces a time-dependent increase of total Rb expression [6]. Increased expression of SDF-1α/CXCR4 in the hippocampus may be associated with enhanced neurogenesis induced by environmental enrichment [11]. AMD3100 reversed the neurogenesis and behavioral recovery promoted by forced limb-use in rats subjected to stroke [12]. These data are examples of the effects of CXCR4 activation that increase nerve cell viability and neurogenesis. However, there are data that show quite the opposite. For example, it was shown that AMD3100 greatly improved neurological scores in mice subjected to brain focal ischemia and reduced inflammatory response, suggesting that CXCR4 activation has an unfavorable effect [13]. Chronic administration of AMD3100 increased survival and alleviated pathology in a superoxide dismutase (SOD1; G93A) mouse model of amyotrophic lateral sclerosis [14]. It was also reported that CXCR4 chemokine receptor signaling mediated pain in diabetic neuropathy [15]. Upregulation of CXCR4 in the dorsal root ganglia and spinal cord contributes to the development and maintenance of neuropathic pain following nerve injury in rats [16]. Systemic inflammation induces anxiety disorder through the CXCR4/SDF-1α pathway [17]. These data show that CXCR4 receptor activation by SDF-1α may increase neuropathic pain, while CXCR4 receptor blockade in brain may have a protective effect on neurons.

On the other hand, neuronal apoptosis—mediated by caspase-3—and oxidative stress contribute to the progression of neurodegenerative diseases [18]. Besides, it is known that Bcl-2 family proteins or specific caspase blockers can inhibit apoptosis [19]. Although antiapoptotic drugs have shown promising results, their use is still limited to preclinical phases, and the efficacy is low due to its inducing drug resistance [18]. Nowadays, the effect(s) of CXCR4 blockade by AMD3100 under normal conditions (in the absence of insults) has not been studied in brain cortical neurons for 7 days in vitro (DIV).

Bcl-2 is an antiapoptotic protein involved in many pathways of programmed cell death, since it regulates the balance between prosurvival Bcl-2-like proteins and proapoptotic Bax-like members [20,21,22,23]. The Bax protein can translocate from cytosol to mitochondria, where it oligomerizes and permeabilizes the mitochondrial outer membrane in order to promote apoptosis. Bax retrotranslocation depends on prosurvival Bcl-2 family proteins, and inhibition of retrotranslocation correlates with Bax accumulation in the mitochondria [24]. Moreover, the upregulation of key apoptotic proteins in human SH-SY5Y neuroblastoma cell line (i.e., Bax, caspase-3/8, and caspase-3/9) contributes to apoptosis by increasing cytochrome c release [25,26,27,28,29,30,31]. A recent study has demonstrated that Bcl-2 antagonist ABT-199 triggers apoptosis and augments ibrutinib-mediated cytotoxicity in CXCR4 wild type and CXCR4 WHIM mutated Waldenstrom macroglobulinemia cells [26]. However, the effect of CXCR4 blockade in brain cortical neurons in the absence of insults is unknown. Since SDF-1α regulates axonal length [32], we have studied, in rat brain cortical neurons in absence of insults, whether CXCR4 blockade by AMD3100 (CXCR4 antagonist) could affect some physiological parameters related to cell viability and mitochondrial function. Thus, several markers like neuronal viability, lactate dehydrogenase (LDH) release, mitochondrial membrane potential (MMP), reactive oxygen species (ROS) production, Bax and Bcl-2 translocation, and cytochrome c release were measured in AMD3100 (200 nM, 24 h)-treated brain cortical neurons and compared to controls (without AMD3100 treatment). We found that, in the absence of insult, CXCR4 blockade induces apoptosis and thus diminishes cell viability of brain cortical neurons without inducing their necrosis.

## 2. Results

### 2.1. Effect of AMD3100 on Cell Viability in Brain Cortical Neurons at 7 DIV (Days in Vitro)

#### Cell Treatment

Rat brain cortical neurons at 7 DIV were treated for 24 h with 200 nM AMD3100. Neurons were washed with phosphate-buffered saline (PBS) and used to test several biochemical parameters described here.

**Figure 1:**
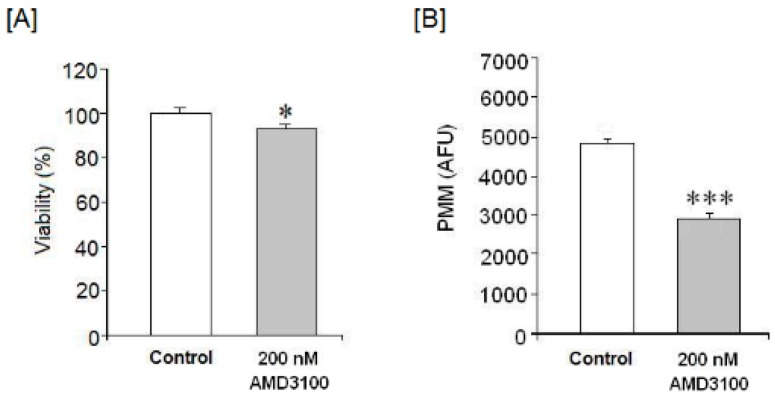
Effect of AMD3100 on (**A**) cell viability and (**B**) mitochondrial membrane potential (MMP). Results are expressed as means ± SEM (standard error of the mean) of two experiments from different cultures, each one performed by triplicate with different batches of cells. The statistical significance was evaluated by *t*-Student test using Sigma Plot 11 software. Differences were considered significant at *p* < 0.05. The asterisks indicate statistical significance as compared to control. * *p* < 0.05 and *** *p* < 0.001. AFU, arbitrary fluorescence units.

AMD3100 led to a loss of neuronal viability in AMD3100-treated brain cortical neurons as compared to untreated controls (*p* < 0.05, Figure 1A). This could mean that CXCR4 blockade by AMD3100 may induce cellular death or a loss of cellular respiratory capacity. 

### 2.2. AMD3100 Altered Mitochondrial Membrane Potential by Hyperpolarization in Rat Brain Cortical Neurons

AMD3100 treatment significantly reduced mitochondrial membrane potential (Figure 1B), suggesting that CXCR4 blockade may induce membrane hyperpolarization, possibly because of some mitochondrial dysfunction.

### 2.3. Effect of AMD3100 on Lactate Dehydrogenase (LDH) Release in Rat Brain Cortical Neurons

**Figure 2:**
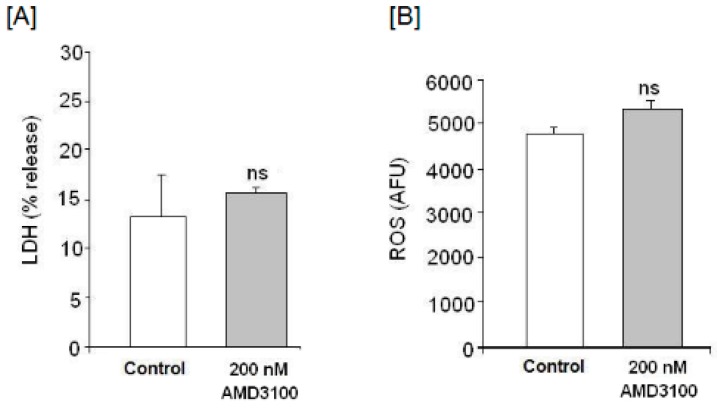
Effect of AMD3100 on (**A**) lactate dehydrogenase (LDH) release; and (**B**) reactive oxygen species (ROS) generation. Results are expressed as means ± SEM of two experiments from different cultures, each one performed by triplicate with different batches of cells. The statistical significance was evaluated by *t*-Student test using Sigma Plot 11 software. Differences were considered significant at *p* < 0.05, statistical significance as compared to control. ns = no significant effect.

Figure 2A shows that AMD3100 treatment does not significantly affect LDH release, which suggests that CXCR4 blockade does not lead to cellular death by necrosis.

### 2.4. Effect of AMD3100 on Reactive Oxygen Species (ROS) Production in Brain Cortical Neurons at 7 DIV

The decrease in cellular viability may mean cellular death or alteration of cellular respiratory capacity subsequent to ROS generation. Therefore, we have evaluated whether CXCR4 blockade by AMD3100 (200 nM) could affect ROS production in cortical neurons as compare to untreated neurons. Figure 2B displays absence of significant changes regarding ROS production in AMD3100-treated cells as compared to controls (*p* > 0.05, no significant effect (ns)).

### 2.5. Effect of AMD3100 on Caspase-9 Activity in Rat Brain Cortical Neurons

Mitochondrial membrane dysfunction may induce release of apoptogenic proteins (from mitochondria to cytosol) to initiate the intrinsic apoptotic pathway. In the cytosol, cytochrome c initiates the caspase-dependent apoptotic pathway, forming the apoptosome and activating caspase-9. Once activated, caspase-9 cleaves and activates caspase-3, which attacks many protein substrates, causing cell death by apoptosis. We have investigated the pattern of cytochrome c localization as well as caspase-9 and caspase-3 enzymatic activities in AMD3100-treated neurons, as compared to controls, by immunoblot.

**Figure 3:**
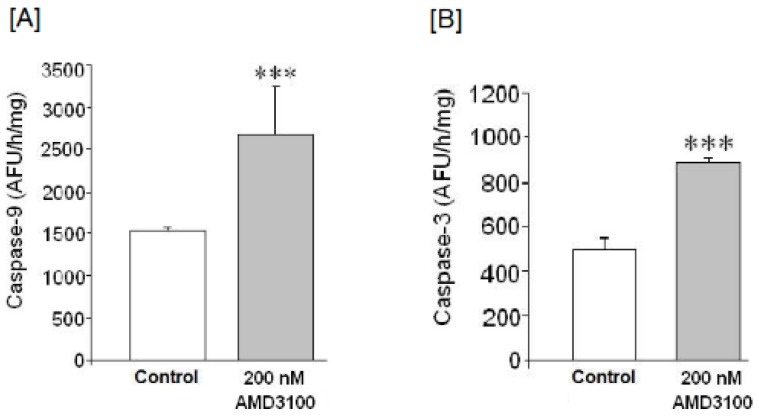
Effect of AMD3100 on (**A**) caspase-9 activity; (**B**) caspase-3 activity. Results are expressed as means ± SEM of two experiments from different cultures, each one performed in triplicate with different batches of cells. The statistical significance was evaluated by *t-*Student test using Sigma Plot 11 software. Differences were considered significant at *p* < 0.05. The asterisks indicate statistical significance as compared to controls, *** *p* < 0.001.

Since AMD3100 mediated cell death without inducing LDH release, we suggest that cell death occurred by apoptosis. The Figure 3A indicates that 200 nM of AMD3100 treatment increased caspase-9 activity as compare to controls (*p* < 0.05).

### 2.6. Effect of AMD3100 on Caspase-3 Activity in Rat Cortical Neurons

Figure 3B show that AMD3100 significantly increased caspase-3 activity. Once we detected that CXCR4 blockade induces apoptosis in AMD3100-treated neurons, we analyzed whether AMD3100 could affect other apoptotic markers like Bax/Bcl-2 protein inducing cytochrome c release.

### 2.7. Effect of AMD3100 on Cytochrome C Release in Rat Brain Cortical Neurons

**Figure 4:**
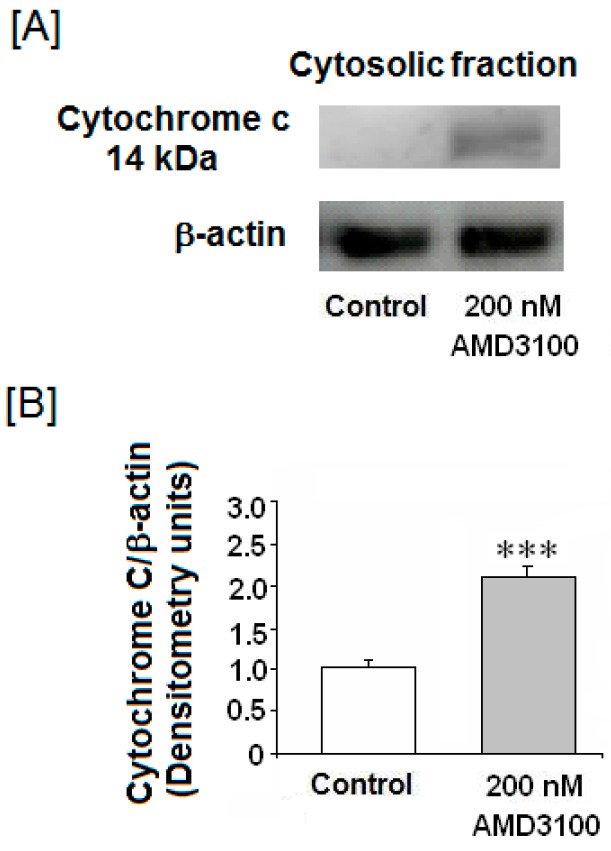
Effect of AMD3100 on cytochrome c release to the cytosol. (**A**) Cytochrome c levels were measured by Western blot; (**B**) average of densitometry from three membranes of three different Western blot analyses. Results are means ± SEM from the three membranes densitometry. The statistical significance was evaluated by *t*-Student test using Sigma Plot 11 software. Differences were considered significant at *p* < 0.05. The asterisks indicates statistical significance as compared to control, *** *p* < 0.001.

In order to confirm whether AMD3100 could induce apoptosis through the activation of the intrinsic pathway, we measured cytosolic cytochrome c release. The cytosolic levels of cytochrome c were significantly higher in AMD3100-treated cortical neurons than in the untreated controls (Figure 4A,B).

### 2.8. Effect of AMD3100 on Cytosolic and Mitochondrial Bax Protein Translocation in Rat Brain Cortical Neurons

**Figure 5:**
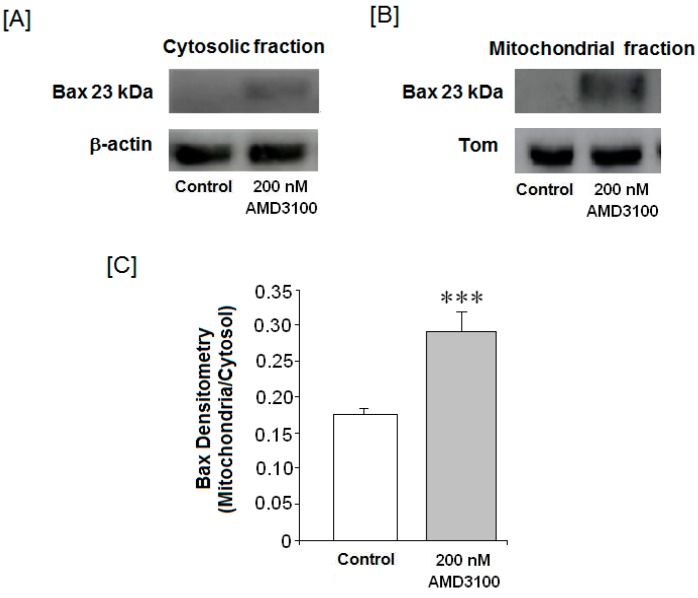
Effect of AMD3100 on Bax protein levels. Analysis of Bax protein levels in the (**A**) cytosol and (**B**) mitochondria by Western blot; (**C**) average of densitometry from three membranes of three different Western analyses. Results are means ± SEM from the three membranes’ densitometry. The statistical significance by *t*-Student test was evaluated using Sigma Plot 11 software. Differences were considered significant at *p* < 0.05. The asterisks indicate statistical significance as compare to control, *** *p* < 0.001.

Bax is a proapoptotic member of the Bcl-2 family of proteins that, under normal conditions, resides in the cytosol, and it is translocated to the outer mitochondrial membrane during apoptosis. Once associated with mitochondria, Bax causes the release of apoptogenic factors from the mitochondria into the cytosol, consequently inducing or propagating the apoptotic cascade. As shown in Figure 5A,B, Bax protein significantly increased in both the cytosolic and mitochondrial fractions in AMD3100-treated neurons as compared to controls. When we evaluated the mitochondria/cytosol Bax ratio, we observed that Bax protein increased within the mitochondrial fraction as compared to its cytosolic levels (Figure 5C). This feature suggests that Bax could have been translocated to the mitochondria.

### 2.9. Effect of AMD3100 on the Mitochondrial Bcl-2 Protein Levels in Rat Brain Cortical Neurons

**Figure 6:**
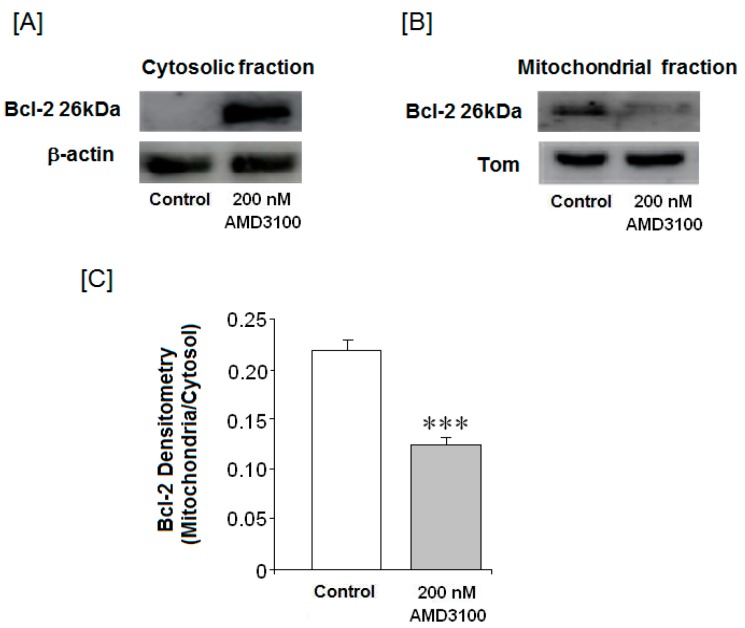
Effect of AMD3100 on Bcl-2 protein levels. Western blot analyses of Bcl-2 protein in (**A**) the cytosol and (**B**) the mitochondria; (**C**) average of densitometry in three different membranes. Results are means ± SEM from the three membranes densitometry. The statistical significance was evaluated by *t*-Student test using Sigma Plot 11 software. Differences were considered significant at *p* < 0.05. The asterisk indicates statistical significance as compare to control. *** *p* < 0.001.

The antiapoptotic Bcl-2 members can inhibit programmed cell death and are associated with cell survival. Under normal conditions, Bcl-2 is in the mitochondria, inducing mitochondrial stability, and providing protection against mitochondrial pore opening. The mechanism involved in this action is not well known. In our study, cytosolic Bcl-2 levels were increased by AMD3100 (200 nM) treatment while mitochondrial Bcl-2 levels decreased (Figure 6A,B). Therefore, the reduced mitochondrial/cytosolic Bcl-2 protein ratio suggests that AMD3100 increases the cytosolic Bcl-2 protein levels (Figure 6A,B).

**Figure 7:**
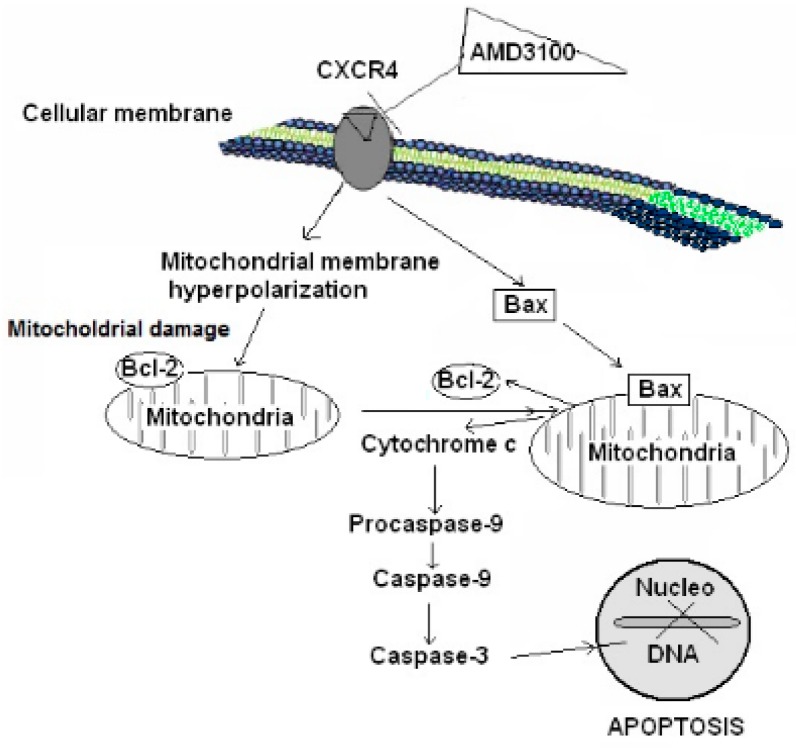
Effects of AMD3100 on rat brain cortical neurons 7 days in vitro (DIV). Chemokine receptor type 4 (CXCR4) blockade induced mitochondrial membrane hyperpolarization and as consequence mitochondrial membrane dysfunction, which led to Bax protein translocation from the cytosol to mitochondria, while Bcl-2 was translocated from the mitochondria to cytosol, increasing the cytosolic Bcl-2 levels. Both events contributed to the opening of mitochondrial pore, leading to cytochrome c release to the cytosol. Finally, this cytochrome c release activated caspase-9, which promoted caspase-3 activation and consequently protein degradation and cell death.

The Figure 7 shows the effect of CXCR4 Blockade on the Survival of Rat Brain Cortical Neurons (summary).

## 3. Discussion

The main aim of the present study was to study whether CXCR4 blockade by AMD3100 could induce cell death in rat brain cortical neurons in the absence of insults. AMD3100 is a known specific antagonist for CXCR4 that does not interact with other chemokine receptors [33]. Our findings have shown that AMD3100 reduced neuronal survival in brain cortical neurons, supporting the notion that the blockade of CXCR4 induces neuronal cell death. In our study, AMD3100 treatment induced a significant mitochondrial membrane hyperpolarization, supporting that CXCR4 blockade could affect mitochondrial integrity. The lack of mitochondrial membrane integrity may be the result of ROS generation. However, this is not the case, since CXCR4 blockade by AMD3100 did not induce ROS formation. The cell death induced by CXCR4 blockade could be mediated by necrosis or apoptosis. Our findings suggest that AMD3100 treatment did not involve LDH release in rat brain cortical neurons. However, AMD3100 induced apoptosis via intrinsic pathway in rat brain cortical neurons since: (1) caspase-3,-9 were activated; (2) Bcl-2 and Bax were translocated (Bax migrated from cytosol to mitochondria, and Bcl-2 migrated from mitochondria to cytosol, conditions which seem necessary to open the mitochondrial pore and to allow the release of cytochrome c); and (3) there was cytochrome c release from mitochondria to cytosol in AMD3100-treated cortical neurons.

Collectively, our findings suggest that CXCR4 blockade in rat brain cortical neurons may lead to the loss of survival, inducing cellular death through intrinsic apoptotic mechanism. However, available data about the CXCR4 receptor behavior are contradictory. Whereas some authors, using different models, have demonstrated that CXCR4 activation may promote reversion of cellular damage induced by several insults [6,11,34], other authors have reported the opposite [15,16,17]. In fact, the activation of CXCR4 chemokine receptor led to detrimental effects in these recent studies [14,15,16,17].

Our findings showing apoptosis in AMD3100-treated brain cortical neurons agree with studies reporting unfavorable effects on neuronal function by blocking CXCR4 chemokine receptor [12,34]. Conversely, other authors have demonstrated beneficial effects on neuronal function by blocking CXCR4 with AMD3100 [13,16,17].

To the best of our knowledge, this is the first study showing that CXCR4 receptor blockade affects neuronal viability of rat brain cortical neurons, as well as several metabolic parameters in the absence of insult.

## 4. Material and Methods

### 4.1. Ethics Statement

Pregnant rats were obtained from Universidad Complutense of Madrid (UCM, Laboratory Animal, License number #ES280790000086). The work was approved by the University Animal Care Committee (CEAE = Committee of Experimental Research and Ethics; form number RD # 53/2013 UCM following Guidelines for the Care and Use of Laboratory; European Council Directive 86/609/EEC). All efforts were made to minimize suffering of animals.

### 4.2. Materials

AMD-3100, tetramethylrhodamine methyl ester (TMRM) and 2,7-dichlorodihydrofluorescein diacetate (2,7 DCF-DA) were purchased from Sigma (Madrid, Spain). Minimum Essential Eagle’s Medium (EMEM, Bio-Whittaker, Walkersville, MA, USA), 2,3-bis-(2-methoxy-4-nitro-5-sulfophenyl)-2*H*-tetrazolium-5-carboxaniline (XTT) test was supplied by (Sigma, Madrid, Spain) and Fetal Calf Serum (FCS) were purchased from Invitrogen, and other chemicals were reactive-grade products from Merck (Darmstadt, Germany).

### 4.3. Methods

#### 4.3.1. Cell Isolation and Culture

Brains from fetal (E19) rats of 19 days gestation were used in the present study. Neurons were grown according to Segal method [35] with minor modifications. The culture cell medium contains EMEM plus 10% FCS, 0.3 g/L glutamine, 3 g/L glucose, 100 U/mL penicillin, and 100 µg/mL streptomycin. Cells were growth at 10^6^ cells/cm well and fixed with 10 µg/mL of poly-d-lysine. Cells were then incubated in a humidified incubator with 5% CO_2_/95% air at 37 °C. After 72 h of incubation, the medium was replaced by fresh medium containing 10 µM of cytosine arabinoside to prevent glial overgrowth. AMD3100 was added at 200 nM concentration (during 24 h) at seven days in vitro (7 DIV). Cell purity was confirmed by cresyl violet staining for neurons while anti-GFAP (glial fibrillary acidic protein) antibody was used to identify astrocytes. Glial contamination was evaluated according to Figueroa et al. [36]. Under these experimental conditions, the glial cells represented 9% ± 3% of the total cell population.

#### 4.3.2. Assessment of Cell Viability by XTT Assay

This assay measures the ability of live metabolically active cells to reduce yellow tetrazolium salt (XTT) to form an orange formazan dye. Thus, this conversion can only occur in living cells. The newly formed formazan dye was directly quantified using a scanning multiwell spectrophotometer at 492 nm of wavelength (reference wavelength 690 nm). The amount of orange formazan formed, as monitored by the absorbance, directly correlates with the number of living cells. Cells were washed with PBS washing buffer. Control and AMD3100-treated (200 nM, 24 h) neurons were incubated with XTT solution (final concentration 0.3 mg/mL) following Kit specifications. After this incubation period, orange dye solution was quantified by spectrophotometry and all results were expressed as percentages and compared with control cells, which is considered as 100%.

#### 4.3.3. Measurement of ROS Formation

To assay ROS formation, 2,7-dichlorodihydrofluorescein diacetate (H2DCF-DA), a nonfluorescent lipophilic reagent, was used in AMD3100-treated cortical neurons and controls (without AMD3100 treatment). H_2_DCF-DA enters the cells, where it is transformed into 2,7-dichlorodihydrofluorescein (H_2_DCF) by intracellular stearases. H_2_DCF is oxidized to fluorescent DCF by hydrogen peroxide. H_2_DCF-DA (5 µM) was added to the cells. After each treatment, the incubation medium was removed and all cells were twice washed with PBS. Finally, fluorescence has been measured in a FL600-BioTek spectrofluorometer (Winooski, VT, USA) with filters of 485/20 nm excitation and 530/25 nm emission. Results are expressed as arbitrary fluorescence units (AFU).

#### 4.3.4. Cellular Death (LDH Assay)

Intracellular enzyme lactate dehydrogenase (LDH) assay is released to the cell culture medium under cell death. Thus, LDH activity detects primary necrotic cell death but could identify “secondary necrosis” due to LDH release from apoptotic bodies (in the absence of phagocytosis) [37]. This activity was determined by spectrophotometric assay following López et al.’s procedure [38]. Briefly, this assay measures the rate of decrement, at 340 nM of absorbance, resulting from the oxidation of NADH:

Pyruvate + NADH → Lactate + NAD^+^

Cortical neurons (6 × 10^5^ cells/well in 24-well plates) were AMD3100-treated (200 nM, 24 h, at 37 °C). Culture medium from all samples were collected. The cells were homogenized by adding 0.1 M KH_2_PO_4_/K_2_HPO_4_ (pH 7.4) and 0.1% of Triton X-100. Homogenates were centrifuged at 13,000× *g* for 10 min. LDH activity was measured in all supernatant as well as in the culture medium. LDH activity release is expressed as percentage as compared to total LDH content (LDH in the supernatant + LDH inside the cells) following this equation: % LDH release = (LDH activity in the medium × 100)/total LDH activity (LDH medium + LDH release).

#### 4.3.5. Mitochondrial Membrane Potential Measurement (MMP)

Changes in the mitochondrial membrane potential were monitored by fluorescent dye tetramethylrhodamine methyl ester (TMRM) assay, according to Tenneti et al.’s 1998 procedure [39]. AMD3100-treated neurons and untreated controls were washed and incubated for 30 min with 500 nM TMRM. After this exposure time, the TMRM was removed and neurons were washed with PBS. Fluorescence was monitored with a FL600-BioTek spectrofluorometer at 530/25 nm excitation and 590/20 nm emission. Fluorescence intensity was expressed as arbitrary fluorescence units (AFU).

#### 4.3.6. Caspase-3 Activity Measurement

AMD3100-treated cortical neurons and controls (without AMD3100 treatment) were washed with PBS and lysed by adding lysis buffer (10 mM Tris-HCl, 10 mM NaH_2_PO_4_/Na_2_HPO_4_, pH 7.5, 130 mM NaCl, 0.5% Triton X-100, 10 mM Na4P_2_O_7_, and 2 mM dithiothreitol (DTT)). Lysates were centrifuged at 13,000× *g* for 10 min. Caspase-3 activity was measured in all supernatants. These supernatants were incubated at 37 °C for 2–6 h in caspase-3 assay buffer (20 mM HEPES, pH 7.5, 10% glycerol, 2 mM DTT containing 20 µM Ac). *N*-acetyl-Asp-Glu-Val-Asp-7-amino-4-methylcoumarin (AcDEVD-AMC). The fluorogenic 7-amino-methylcoumarin (AMC) released from Ac-DEVD-AMC was monitored using a spectrofluorometer BioTek FL 600 (Winooski, VT, USA), at excitation wavelength of 360/20 nm and an emission wavelength 460/20. Enzymatic activity is expressed as arbitrary fluorescence unit after 1 h per µg protein (AFU/h/µg protein). The protein concentrations of cell extracts were determined by the Bradford [40] method using bovine serum albumin as standard.

#### 4.3.7. Caspase-9 Activity

Caspase-9 activity was measured following the same described procedure for caspase-3 but using selective substrates (Ac-LEHD-AFC-(Leu-Glu-His-Asp-(7-amino-4-trifluoromethyl-coumarin), AFC)).

### 4.4. Preparation of Cell Lysates (Subcellular Fractionation)

Cortical neurons were harvested by scraping into hypotonic buffer (10 mM Tris-HCl; pH 7.4), 1% SDS, 1 mM sodium vanadate, and 0.01% protease inhibitor cocktail (all from Sigma Aldrich, Madrid, Spain) and incubated on ice. The lysates were centrifuged at 800× *g* for 5 min at 4 °C to remove the nuclei and cellular debris (Centrifuge 5804R, Eppendorf, Hamburg, Germany). The resulting supernatants were further centrifuged at 15,000× *g* for 15 min at 4 °C to obtain the supernatant fraction (cytosol) and a pellet containing mitochondria, which was resuspended in lysis buffer (50 mM Tris, 150 mM NaCl, 10 mM EDTA, 1% Triton X-100). Both cytosolic and mitochondrial fractions were aliquoted and stored at −80 °C for further biochemical analysis. The protein concentration was determined by the DC Protein Assay Kit (Bio-Rad; Madrid, Spain). Bovine serum albumin was used in a concentration range of 0–2 µg/µL as standard.

### 4.5. Protein Evaluation of Apoptotic Markers by Western Blot

The protein cytochrome *c*, Bcl-2 and Bax were assessed in the cytosolic and/or mitochondrial fractions of cortical neurons extracts in the presence/absence of AMD3100 treatment (200 nM, 24 h). Equal amounts of protein in a volume of 30 µL were separated on a 15% polyacrylamide gel (SDS-PAGE). These gels were transferred to a polyvinylidene fluoride (PVDF) membrane (GE Healthcare, Madrid, Spain). All membranes were blocked by adding 5% nonfat dry milk for 1 h at room temperature. The membranes were incubated overnight at 4 °C with the appropriate primary antibodies: goat polyclonal anti-cytochrome c for immunodetection (1/500) (sc-7159, Santa Cruz Biotechnology, Quimigen, Madrid, Spain), rabbit polyclonal anti-Bax (1/250) (sc-493), and anti-Bcl-2 (1/250) (sc-492) (Santa Cruz Biotechnology, Quimigen, Madrid, Spain) followed by incubation with peroxide-conjugated secondary antibodies for 1 h at room temperature (Santa Cruz Biotechnology, Quimigen, Madrid, Spain). Blots were developed by enhanced chemiluminescence (ECL select; GE Healthcare, Madrid, Spain) following manufacturer’s instructions. Anti β-actin (sc-47778) and anti-Tom-20 (sc-11415) antibodies (Santa Cruz Biotechnology, Quimigen, Madrid, Spain) were used as loading control for cytosolic and mitochondrial extracts, respectively.

### 4.6. Statistical Analysis

Data are presented as means ± SEM of three independent experiments using different cell cultures, each one performed by triplicate. Data were analyzed by *t*-Student test using Sigma Plot 11 software. Differences were considered significant at *p* < 0.05.

## 5. Conclusions

1. Under normal conditions, CXCR4 blockade by AMD3100 decreased cellular viability independently of neuronal necrosis or ROS formation in rat brain cortical neurons at 7 DIV.

2. AMD3100 altered membrane mitochondrial potential by inducing hyperpolarization and promoted apoptotic events through intrinsic pathway of apoptosis by activating caspase-9/-3.

3. The CXCR4 blockade in cortical neurons induced apoptosis through cytochrome c release not only by increasing cytosolic Bcl-2 protein levels, but also promoting Bax translocation to the mitochondria in AMD3100-treated neurons.
